# Transfer of Copper (Cu) in the Soil–Plant–Mealybug–Ladybird Beetle Food Chain

**DOI:** 10.3390/insects13090761

**Published:** 2022-08-24

**Authors:** Xingmin Wang, Mengting Zhang, Huiyi Cao, Mudasir Irfan Dar, Shaukat Ali

**Affiliations:** 1 Key Laboratory of Bio-Pesticide Innovation and Application, College of Plant Protection, South China Agricultural University, Guangzhou 510642, China; 2 Engineering Research Center of Biological Control, Ministry of Education and Guangdong Province, South China Agricultural University, Guangzhou 510642, China; 3 Plant Ecology Laboratory, Department of Botany, Aligarh Muslim University, Aligarh 202002, India

**Keywords:** heavy metals, copper, solanum melongena, *Ferrisia virgata*, *Nephus ryuguus*

## Abstract

**Simple Summary:**

This study explains the transfer of Cu through a food chain consisting of a plant (eggplant), mealybug (*Ferrisia virgata*) and the latter’s predator (*Nephus ryuguus*). The Cu transfer was effectively reduced in the root–shoot–mealybug food chain.

**Abstract:**

Copper, an essential trace element, is vital for living organisms’ survival; however, despite its importance, an excessive amount of this micro-nutrient can cause harmful effects to plants and animals. The present study investigates Cu bio-transfer across multi-trophic food chain comprising soil (spiked with various concentrations of Cu), eggplant (*Solanum melongena*), mealybug (*Ferrisia virgata*), and ladybird (*Nephus ryuguus*). Soils were spiked with Cu at rates of 0, 100, 200, 400, and 800 mg/kg (*w*/*w*). A dose-dependent increase in the levels of Cu in plant, mealybug, and ladybird was observed in response to Cu contents of soil. Different Cu amendment caused a significant reduction in the average root and shoot dry weights per plant as well as the average body weights of *F. virgata* and *N. ryuguus*. Our findings affirmed the importance of additional research to explain the processes involved in the bio-transfer of copper across the food chain.

## 1. Introduction

Heavy metals make their entry into the ecosystem by natural as well as anthropogenic means [1,2]; however, ecological risk is posed due to the continued addition of heavy metals to soil through transportation, mining, military operations, industrialization, fertilizer application (both mineral and organic) and pesticides in crops [3]. This leads to the accumulation of trace metals in soil for longer periods of time. The subsequent absorption of trace elements by plants may pose a threat to other organisms of higher trophic levels even if accumulated by plants at sub-phytotoxic levels [4]. Copper (Cu) plays essential physiological and biochemical roles in plants [5,6], but excessive levels can cause damage to environmental health. The measurements of total copper concentrations in the environment (i.e., in surface water, sediments, soils, etc.) cannot be used to predict risks to organisms. Only a small portion of the total amount of copper is bioavailable to organisms and is, thus, potentially toxic. The bioavailability of copper is controlled by the environment’s local chemistry and the mutual interactions of these chemicals and copper with each organism. Higher concentrations of Cu (60–150 mg/kg) can affect the growth and yield parameters of plants, such as the germination of seeds, root and shoot growth rate, and yield [7].

Insects that feed on plants are considered one of the important connecting links to transfer energy from plants to organisms of higher trophic levels in terrestrial ecosystems [8]. Therefore, phytophagous insects may act as carriers of trace metals, translating to higher trophic levels in environments polluted with trace metals [9]. The tendency of metal transfer and bio-accumulation in different ecosystems depends on the biochemistry of metal and the metal regulating the physiology of the organism involved in the food chain [10]. Studies have shown the negative effects of copper formulations on different insect species. Crawford et al. [11] observed deleterious effects of high Cu concentrations on the growth, development and reproduction of *Aphis fabae* (Homoptera: Aphididae).

Predatory arthropods are one of the integral components of natural environments [12]. Within agro-ecosystems, these predators perform valuable functions, such as controlling insect pests [10]. The efficacy, life table, and physiology of predatory arthropods may be adversely affected when they feed on prey polluted with heavy metals [12,13,14]. Mani et al. [15] observed reduced life periods and egg laying by *Leptomastix dactylopii* (Hymenoptera: Encyrtidae) in response to being fed a copper-contaminated diet. Lapointe et al. [16] reared *Diaprepes* root weevil, *Diaprepes abbreviates,* on an artificial diet containing four concentrations of copper and observed negative effects of copper on insect development and survival from hatching to 4 weeks of age. The behavior of Cu in a plant–insect–chicken food chain was demonstrated by Zhuang et al. [4]; however, in plant–insect food chains, Cu transfer has been examined only up to two trophic levels [11]. Therefore, there is a dearth of evidence about the behavior of this metal in higher trophic levels. This study was performed to examine the bio-transfer of Cu across a muti-trophic food chain. Eggplant (*Solanum melongena*) was selected due its tolerance to environmental stress, higher plant weight and usage in everyday human diet [9]. *Ferrisia virgata* (Cockerell) (Hemiptera: Pseudococcidae) was selected as it causes severe damage to different vegetables [17]. We chose *Nephus ryuguus* (Kamiya) (Coleoptera: Coccinellidae) as the organism for third trophic level because it is the dominant predatory natural enemy of mealybugs [18].

Our major aims were to: (a) quantify the amount of Cu transferred from the soil to the food chain via one phytophagous insect species and one herbivorous predator; (b) study the possible enhanced or reduced transfer of Cu from one level to another in the food chain; and (c) assess the changes in dry weight of the three species in response to different Cu concentrations.

## 2. Materials and Methods

### 2.1. Chemical and Reagents

The chemicals and reagents utilized in this study were supplied by Genstar Chemicals, Shenzhen, China.

### 2.2. Insect Cultures

*Ferrisia virgata* colonies were reared according to Qin et al. [19]. In short, reproductively active females (*n* = 100) of *F. virgata* were cultured on eggplants for three successive generations under laboratory conditions (26 ± 1 °C, 75 ± 10% R.H.) and a light and dark photoperiod (14 h:10 h). Mated *F. virgata* females (second generation) were placed in rearing cages (90 × 63 × 50 mm) for egg laying. The freshly hatched 4th instar nymphs were utilized for the whole experiment.

*Nephus ryuguus* colonies were reared on *F. virgata* for several successive generations under laboratory conditions (26 ± 1 °C, 75 ± 10% R.H.) and a light and dark photoperiod (14 h:10 h).

### 2.3. Experimental Design

For this study, sandy loam soil (having no cultivation history and a background Cu concentration of 10 mg/kg; supplied by Green Farms agricultural services Co. Ltd. Guangzhou, China) was divided into five equal parts as described by Du et al. [14]. For soil Cu spiking, copper sulfate pentahydrate (CuSO_4_·5H_2_O) was used. Copper sulfate pentahydrate was dissolved in water to obtain a standard Cu solution formulation with a concentration of 2000 ppm. Four soil samples were spiked with a stock solution of CuSO_4_·5H_2_O (the solutions of metal and soil were thoroughly mixed) to achieve copper-spiked soils of different concentrations (T_1_ = 100, T_2_ = 200, T_3_ = 400 and T_4_ = 800 mg/kg), according to Kabata-Pendias and Pendias [3], while the fifth sample was not spiked with CuSO_4_·5H_2_O, considered the control (T_0_). There were three replications of all treatments and control. The soils from different treatments were individually added to plastic pots (Ø 250 mm). The pots were left for an equilibrium period of three weeks and soil samples (10 g) were collected after 3 months to analyze soil properties. After 3 months of soil spiking, *Solanum melongena* (Dafeng cultivar obtained from Guangdong Academy of Agricultural Sciences, Guangzhou, China) seedlings (2 weeks old) were transferred to plastic pots (one seedling/pot) having soil subjected to different Cu treatments. The leaching of Cu was controlled by placing a plastic plate beneath the pots. The pots were placed in the greenhouse using a completely randomized design. De-ionized water was added to the pots as per requirement of plants for the maintenance of normal growth. Plants were allowed to grow until the flowering stage (about 6 weeks) and plant samples were collected for Cu analysis. At the flowering stage, three hundred mealybug individuals (4th instar nymphs) were transferred (from rearing colonies) to each plant with a camel hairbrush and were allowed to feed for 3 weeks. After 3 weeks of feeding, mealybugs were grouped into two sets. The first set was utilized for Cu analysis and the second was used to feed the predatory beetle *Nephus ryuguus.*

The mealybugs harvested from pot cultures of eggplants were used for feeding experiments following the method of Green et al. [20], briefly described as follows: 4th instar larvae of *N. ryuguus* (75 individuals) separated from laboratory culture were divided into five equal treatment groups and each individual was randomly assigned to mealybugs harvested from pot cultures of eggplants in Petri dishes (Ø 9 cm) lined with a moist filter paper. The whole experimental setup was incubated at 25 ± 2 °C. Fresh mealybugs were added to the Petri dishes for *N. ryuguus* larval feeding until all the beetles had pupated (2 weeks). The number of mealybugs consumed by ladybird larvae was recorded every 24 h. The adult beetles were weighed on emergence and then left at −20 °C until Cu quantification.

### 2.4. Preparation of Samples for Cu Content Assays

The samples of soil (*n* = 15) collected at 150 mm depth were air-dried, crushed, and passed via a sieve with mesh (2 mm). The contents of organic carbon and total nitrogen were quantified according to Allison [21] and Dar et al. [22]. The pH of soil samples (soil water suspension of 1:2.5 *w*/*v*) was measured by using a pH meter (Model: PHSC-3, INESCA instruments, Shanghai, China).

The Cu fractions were extracted from the soil sample (1 g) in a 20 mL digestion mixture (HNO_3_/H_2_O_2_) at a ratio of 6:1 at 80 °C. Upon the completion of digestion, the solution was cooled and filtered using filter paper (Whatman No.42) followed by dilution to a final volume of 50 mL with double-distilled water (ddH_2_O) as a diluent. The plant available Cu concentration was extracted by DTPA extraction (extraction with DTPA + CaCl + triethanolamine) following Lindsay and Norvell [23].

After two months, eggplants were harvested carefully and cleaned out thoroughly, once with tap water and twice with ddH_2_O, to remove soil particles on the plants’ surfaces. Different plant regions (roots, shoots and leaves) were separated. The plant samples were placed at 70 °C until a constant weight. The dried sample of 0.3 g was digested in a 10 mL digestion mixture (HNO_3_/H_2_O_2_) at a ratio of 6:1 at 80 °C until the solution was entirely transparent. Upon the completion of digestion, the solutions were allowed to cool and filtered using filter paper (Whatman No.42). The dilution of filtered solution was carried out with double-distilled water (ddH_2_O) to a final volume (50 mL).

Sub-samples of mealybug and beetles were rinsed once with tap water and then with ddH_2_O. Later on, mealybug sub-samples (20 mg) and individual ladybird beetles (5 adults) were digested in a digestion solution of 2 mL (HNO_3_/H_2_O_2_) at a ratio of 6:1 at 80 °C. The dilution of digested solution was carried out with ddH_2_O to a required volume (5 mL). Copper contents in all samples were measured using an electro-thermal atomic absorption spectrometer, AA6300C (Shimadzu, Kyoto, Japan).

### 2.5. Statistical Analysis

One-way ANOVA and Welch’s *t*-test were used to predict the properties of soil (physical and chemical) and data are presented as mean ± S.E by using the REGW (Ryan–Einot–Gabriel–Welsch) multiple-range test. The Cu transfer co-efficient was calculated by following the equation

Cu transfer co-efficient = Cu concentration in level B/Cu concentration in level A where level A represents a lower level of the food chain and level B a higher level of the food chain.

One-way ANOVA was used to analyze Cu transfer and dry weight data, and Tukey’s HSD test was used to compare the means (at 5% level of significance). All statistical analyses were performed using SAS version 8.01 for windows [24].

## 3. Results

### 3.1. Effect of Copper Treatments on Soil Properties

**Figure 1:**
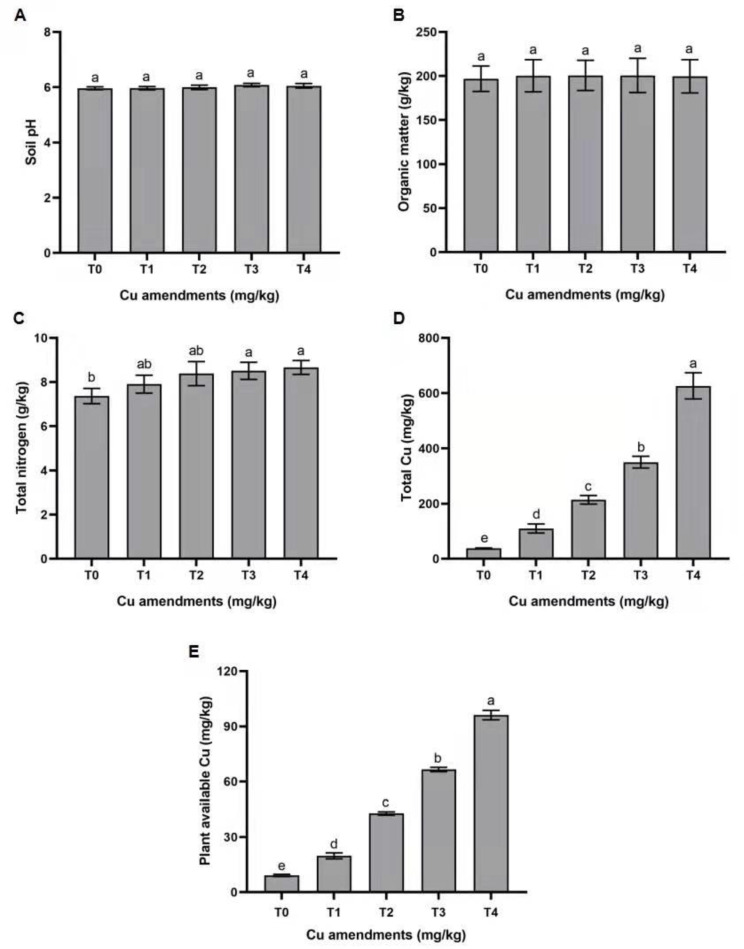
Variation in chemical properties and Cu concentrations (total and extractable) of soil treated with different Cu concentrations. (**A**) Soil pH; (**B**) organic matter (g/kg); (**C**) total nitrogen (g/kg); (**D**): total Cu (mg/kg); (**E**): plant available Cu (mg/kg). T0= control, T1 = 100, T2 = 200, T3 = 400 and T4 = 800 mg/kg. Each value is the mean of three replicates ± S.E. Bars with different letters are significantly different from each other (Tukey’s *p* < 0.05).

No significant differences were observed between Cu-spiked and control soils in terms of pH and the organic matter content of soil (Figure 1A, B). The total soil nitrogen contents differed significantly between Cu-spiked soils and controls (F_4,10_ = 5.05; *p* = 0.017). The Cu-spiked soil treatments using higher concentrations (T_3_ and T_4_) had significantly higher total N contents when compared with the control (T_0_) (Figure 1C). The Cu-spiked soils had significantly higher total Cu contents as well as plant available (mobile) Cu contents when compared with control (Figure 1D, E).

### 3.2. Effects of Cu Soil Spiking on Soil to Root Cu Transfer and Root Dry Weight

**Figure 2:**
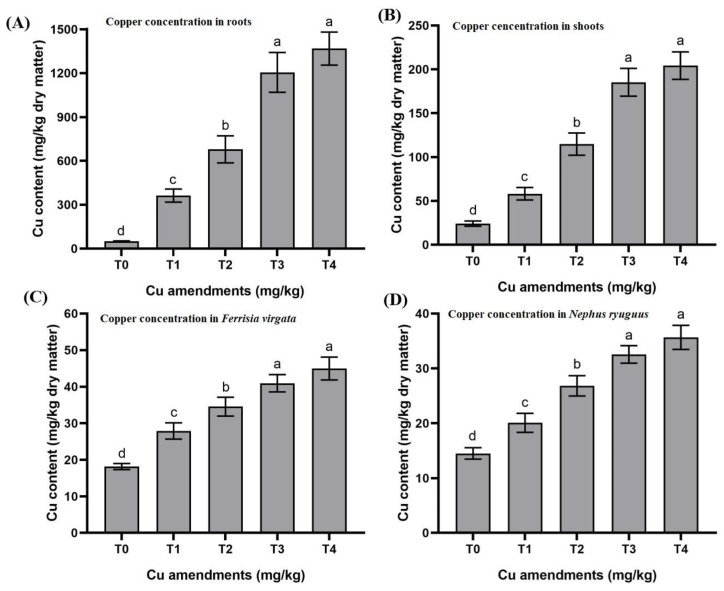
(**A**–**D**) Copper concentrations (mg/kg dry matter) accumulated in plants, mealybug and newly emerged ladybird. T0= control, T1 = 100, T2 = 200, T3 = 400 and T4 = 800 mg/kg. Each value is the mean of three replicates ± S.E. Bars with different letters are significantly different at *p* < 0.05.

The concentration of Cu in roots differed significantly among Cu-spiked and control soils (F_4,10_ = 538.83; *p* < 0.001). For eggplant, the highest root Cu concentration was observed in T_4_, and the lowest root Cu concentration was observed in T_0_, with average values of 93.85 ± 2.50 mg/kg dry matter and 7.45 ± 0.27 mg/kg dry matter, respectively (Figure 2A).

The Cu transfer coefficient from soil to eggplant roots varied across the soil treatments, ranging from 0.15 to 0.20 for total Cu in soil to roots and 0.82 to 0.98 for plant available Cu fraction (DTPA) to root (Table 1).

**Figure 3:**
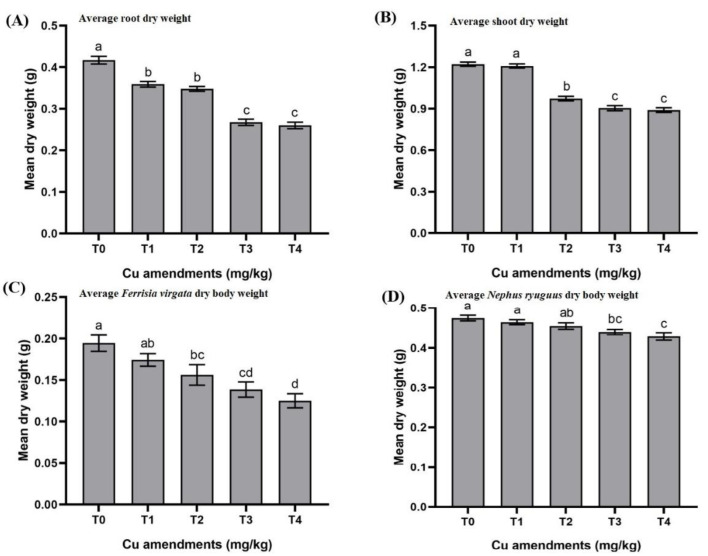
(**A**–**D**) Changes in mean dry weights of different trophic levels of food chain in response to soil amendment with different Cu concentrations. T0 = control, T1 = 100, T2 = 200, T3 = 400 and T4 = 800 mg/kg. Each value is the mean of three replicates ± S.E. Bars with different letters are significantly different at *p* < 0.05.

The root dry weight differed significantly between the Cu-spiked soils and control (F_4,10_ = 233.19; *p* < 0.001). The highest root dry weight was observed for plants from T_0_, with an average value of 0.42 ± 0.01 g per plant, whereas the lowest root dry weight (0.26 ± 0.01 g per plant) was observed for plants from in T_4_ (Figure 3A).

### 3.3. Effects of Cu Soil Spiking on Root to Shoot Cu Transfer and Shoot Dry Weight

The concentration of Cu in shoots differed significantly among plants grown in different Cu-spiked soils and control (F_4,10_ = 91.01; *p* < 0.001). T_4_ had the highest Cu concentration in eggplant shoots, with an average value of 92.65 ± 1.38 mg/kg dry matter. The lowest Cu content of shoots was found in T_0_, with an average value of 6.95 ± 0.29 mg/kg dry matter (Figure 2B).

Copper transfer coefficients from roots to shoots for different treatments ranged between 0.93 and 0.99 and were always lower than 1 (Table 1).

The shoot dry weight per plant was significantly different among different Cu treatments and control (F_4,10_ = 30.26; *p* < 0.001). The highest shoot dry weight was observed for plants from T_0_, with an average value of 1.22 ± 0.01 g per plant, whereas the lowest root dry weight (0.89 ± 0.01 g per plant) was observed for plants from in T_4_ (Figure 3B).

### 3.4. Effects of Cu Soil Spiking on Shoots to Mealybug Cu Transfer and Mealybug Dry Body Weight

The concentrations of Cu in mealybug bodies increased significantly with different Cu treatments (F_4,10_ *=* 27.64; *p* < 0.001). The lowest Cu contents were found in mealybugs fed on T_0_ treated eggplants, with an average value of 6.89 ± 0.46 mg/kg dry matter. The maximum amount of Cu (64.96 ± 1.80 mg/kg dry matter) accumulated in mealybugs feeding on plants from T_4_ (Figure 2C).

The Cu transfer coefficient from eggplant shoots to mealybugs remained below one in all Cu treatments and showed a decreasing trend (from 0.99 to 0.70) from the control to the highest Cu treatments (Table 1).

Different Cu amendments caused a significant reduction in the average body weight of mealybugs (F_4,10_ = 24.63; *p* < 0.001). The highest body weight of mealybug (0.19 ± 0.01 mg per individual) was observed for T_0_, whereas the mealybug dry weight (0.12 ± 0.01 mg per individual) was observed for plants in T_4_ (Figure 3C).

### 3.5. Effects of Cu Soil Spiking on Mealybug to Cu Transfer and Beetles’ Dry Body Weight

The Cu concentration in predatory beetles increased significantly in different Cu treatments (F_4,10_ = 36.98; *p* < 0.001). The highest Cu contents (45.66 ± 1.26 mg/kg dry matter) were observed in *N. ryuguus* feeding on mealybugs from T_4_. The lowest Cu contents were observed in the bodies of *N. ryuguus* fed on mealybugs from control treatment with an average value of 4.49 ± 0.60 mg/kg dry matter (Figure 2D).

The coefficients of Cu transfer between mealybugs and *N. ryuguus* on eggplant varied between different Cu amendment rates, ranging from 0.65 to 0.93 (Table 1).

The average body dry weight of *Nephus ryuguus* differed significantly between different treatments (F_4,10_ = 18.12; *p <* 0.001). The highest body weight of *N. ryuguus* was observed for T_0_ with an average value of 0.48 ± 0.01 mg per individual, whereas the lowest *N. ryuguus* body weights (0.43 ± 0.01 mg per individual) were observed for T_4_ (Figure 3D).

## 4. Discussion

### 4.1. Effects of Cu on Soil Properties

Different soil variables, such as soil pH, soil organic matter, mineral composition and water regimes, control the bio-availability of Cu in soil. Our findings revealed that different copper concentrations did not correlate with total organic matter content, which is in line with previous findings [3]. Although, the plant available (mobile) concentration of Cu showed a slight increase. However, for all treatments, a significant increase above the permissible limits was observed (20–100 mg kg^−1^) [3]. The higher values of plant available Cu observed here might be related to the interaction of copper with soil organic matter as Cu binds with organic soil constituents followed by retention in soil through cation exchange [25].

### 4.2. Copper Transfer from Soil to Roots

Soil modification with various copper concentrations caused Cu accumulation in eggplant roots. The concentrations of Cu along with the coefficients of Cu transfer from soil to roots were higher as compared to other points in the food chain. Copper concentrations in eggplant roots were higher than the copper critical limits (60–150 mg/kg), as reported by Kabata-Pendias and Pendias [3]. Our findings are in accordance with an earlier study by Bunzl et al. [26], who noticed an increase in the concentrations of Cu in the roots of bean, lettuce, carrot, and celery after growing these vegetables in Cu-contaminated soil. Higher Cu concentrations in eggplant roots caused a reduction in root dry weight. Dresler et al. [7] observed an up to 80% reduction in root dry weight in response to higher Cu concentrations (50–100 µM). Kloke et al. [27] reported the growth depression of plant tissues at 15–20 mg/kg Cu, while Macnicol and Beckett [28] showed a 10% reduction in plant yield within the Cu range of 10–30 mg/kg. The reduction in root dry weight could be due to tissue damage or a change in membrane permeability, causing the leakage of ions and solutes from root cells [29,30].

### 4.3. Copper Transfer from Roots to Shoots

In eggplant, the translocation rate of copper in response to Cu soil amendments (from roots to shoots) was concentration-dependent. However, copper transported from roots to shoots was lower than that from the soil to the root. As expected, the copper concentrations translocated from roots to shoots were decreased as compared with soil to root transfer. Our findings are in accordance with the results of Damrongsiri and Chotipong [31], who reported lower Cu accumulation in rice straw compared to roots in the following order: roots > straw > grain > husk. Our results are also consistent with the findings of Yan et al. [32], who observed higher Cu accumulation in the roots of *Poaceae* species as compared to aerial parts. The lower rate of the translocation of Cu from root to shoot is probably due to sequestration/detoxification through chelation in the cytoplasm or its movement into the vacuoles of root cells [33]. Thus, the sequestration of Cu in roots acts as a first checkpoint for translocation towards aerial parts. The upward transfer of Cu from the roots to shoot regions can directly affect the aerial plant parts, causing reduced photosynthesis along with cellular metabolism and as a result retarded plant growth [34,35]. Cu toxicity can also affect hydrolytic enzyme activities, leading to reduced nutrient transport to aerial parts, which ultimately leads to reduced plant growth [36,37]. Higher Cu concentrations in eggplant shoots caused a reduction in shoot dry weight. This decrease in shoot biomass can be correlated with disturbed metabolic process, low photosynthetic response, and the absorption of essential minerals under heavy metal stress [38].

### 4.4. Copper Transfer from Shoots to Mealybug and from Mealybug to Adult Beetles

Final Cu concentrations detected in *F. virgata* and *N. ryuguus* bodies were higher than the accumulation of other metals (Pb and Cd) in the bodies of mealybug (*Dysmicoccus neobrevipes*) and ladybird (*Cryptolaemus montrouzieri*) in previous studies [8,9]. This higher accumulation of Cu at insect levels (mealybug and predatory beetles) of the food chain can be related to essential metabolic requirements of Cu since insects have developed the ability to store this metal for the maintenance of homeostasis [20]. The higher concentrations of Cu caused a significant reduction in the mean dry weight of mealybugs (*F. virgata*) and adult beetles (*N. ryuguus*). This reduction in the body weight of predatory beetles can affect their life history parameters and may incur significant energy costs through the production of detoxification enzymes [39]. Our findings are related to a new and relatively unexplored area of biological control research, with important implications for our understanding of agricultural food web interactions.

## 5. Conclusions

Our study showed the bio-transfer of Cu along a multi-trophic soil–plant–mealybug–ladybird food chain. Significant differences were observed in the concentrations of copper over various components of the food chain. A higher bio-transfer of Cu (per unit) was observed for soil–root, shoot–mealybug and mealybug–ladybird. In spite of Cu being an essential trace metal for plant and insect growth, a precautionary approach is necessary at higher trophic levels of the food chain because higher copper concentrations can harm the organisms. Therefore, future research in biological and physiological studies is needed to explore the aforementioned effects under field conditions. Our findings are related to a new and relatively unexplored area for biological control research, with important implications for our understanding of agricultural food web interactions.

## Figures and Tables

**Table 1:** Coefficients of Cu transfer between various components of soil–plant–mealybug–ladybird after soil amendment with copper.

Cu Treatments (mg/kg Soil)	Total Soil–Root	Extractable Soil–Root	Root–Shoot	Shoot– *Ferrisia Virgata*	*Ferrisia Virgata*– *Nephus Ryuguus*
Control	0.20	0.82	0.93	0.99	0.65
100	0.17	0.96	0.93	0.96	0.93
200	0.20	0.98	0.96	0.85	0.78
400	0.18	0.94	0.97	0.73	0.79
800	0.15	0.98	0.99	0.70	0.70

## Data Availability

The raw data supporting the conclusions can be made available by the corresponding author on request.
